# Microbial Contamination Detection in Water Resources: Interest of Current Optical Methods, Trends and Needs in the Context of Climate Change

**DOI:** 10.3390/ijerph110404292

**Published:** 2014-04-17

**Authors:** Aude-Valérie Jung, Pierre Le Cann, Benoit Roig, Olivier Thomas, Estelle Baurès, Marie-Florence Thomas

**Affiliations:** 1 School of Environmental Engineering (EME), Campus de Ker Lann, Avenue Robert Schuman, Bruz 35170, France; E-Mail: marieflorencethomas@ecole-eme.fr; 2 EHESP Rennes, Sorbonne Paris Cité, Avenue du Professeur Léon Bernard-CS 74312, Rennes Cedex 35043, France; E-Mails: pierre.lecann@ehesp.fr (P.L.C.); benoit.roig@unimes.fr (B.R.); olivier.thomas@ehesp.fr (O.T.); estelle.baures@ehesp.fr (E.B.); 3 INSERM, UMR IRSET Institut de Recherche sur la Santé l’Environnement et le Travail - 1085, LERES, Rennes 35043, France; 4 European University of Brittany (UEB), 5 Boulevard Laënnec, Rennes 35000, France; 5 Deparment of Sciences, Nîmes University, Rue du docteur George Salan, Nîmes 30000, France

**Keywords:** optical methods, heavy rainfall, colloids, turbidity, pathogens

## Abstract

Microbial pollution in aquatic environments is one of the crucial issues with regard to the sanitary state of water bodies used for drinking water supply, recreational activities and harvesting seafood due to a potential contamination by pathogenic bacteria, protozoa or viruses. To address this risk, microbial contamination monitoring is usually assessed by turbidity measurements performed at drinking water plants. Some recent studies have shown significant correlations of microbial contamination with the risk of endemic gastroenteresis. However the relevance of turbidimetry may be limited since the presence of colloids in water creates interferences with the nephelometric response. Thus there is a need for a more relevant, simple and fast indicator for microbial contamination detection in water, especially in the perspective of climate change with the increase of heavy rainfall events. This review focuses on the one hand on sources, fate and behavior of microorganisms in water and factors influencing pathogens’ presence, transportation and mobilization, and on the second hand, on the existing optical methods used for monitoring microbiological risks. Finally, this paper proposes new ways of research.

## 1. Introduction

In the context of international regulations, the contamination of water bodies by organic micropollutants is the subject of constant interest and is always under investigation. Although at the origin of numerous outbreaks of gastrointestinal diseases and public health concerns, microbial contamination is rarely considered [1], even if some studies have shown consistent and significant association between heavy rainfall events and waterborne disease outbreaks [2,3] and climate change consequences [4]. A lack of studies concerning waterborne diseases correlated with extreme events (such as droughts and floods) has recently been mentioned by a review of reviews [5]. Despite a regulatory framework, microbial quality is often a source of impairment of the compliance of drinking water supply [6], in particular for small scale water systems (SSWS) [7,8] and private water supplies [9,10,11]. Additionally, water quality is known to be affected by increased microbial pollution under extreme weather conditions (climate change) and requires more systematic studies [12,13]. Monitoring objectives consist in directly targeting the sources of contamination, by using simple and rapid indicators but are mainly focused on parameters such as faecal bacteria (*E. coli* or *Enterococci*). Enteric viruses, that play a major role in waterborne diseases, are rarely investigated due to the detection limits of commonly applied methods [14,15]. Consequently, even when complying with microbiological water quality standards, the endemic part of waterborne acute gastroenteritis (AGE) may vary from 0 to 40% [16,17] excluding the smallest supply water systems (<500 inhabitants) for which the syndromic surveillance is ineffective. 

For many years, analytical approaches have been developed for knowledge improvement concerning microorganisms’ nature and origins. More recently, different studies have shown the importance of an ecological (sediment and aquatic plants role) approach [18,19,20,21,22,23]. Although PCR is often used in microbial source tracking, it has not found widespread application in microbial monitoring programs due to its targeted approach for specific microbial genera or species [24,25]. MST tools proposed for the identification of the origin of faecal pollution are well documented in the literature [21,26,27,28,29,30]. Except for these methods and the conventional bacterial culture (standardized), some alternative ones are available, such as methods based on the exploitation of the optical properties of water. The interest in optical methods for microbial detection may be justified by the optical effects (mainly absorption and light diffusion) of microorganisms often adsorbed on colloids or particles. The effect of interaction between light and matter can be exploited to detect bacteria or viruses either as free particles or attached to organo-mineral complexes capable of modifying the optical properties of surfaces. 

This review focuses on: (1) sources, fate, behavior of microorganisms in water and factors influencing pathogens’ presence, transport and mobilization, and (2), on optical methods for microbial contamination monitoring. 

## 2. Sources, Fate and Behavior of Microorganisms in Water

The microbial contamination of water is often of faecal nature related to humans (water sewage treatment plants, combined sewage overflow (CSO), non-collective sewage systems), domesticated animals (manure spreading, pit stock overflow), or wildlife. The main origins of microbial contamination of natural aquatic resources are discharges of water treatment plants, decontamination stations, hospitals, industries considered as point sources, *etc*. Correlation between pathogens concentrations and urban activities is well documented (e.g., [31,32]). On the other hand, diffuse sources (slurry, manure, sludge application…) may also be considered. The abundance and importance of pathogens in water depend on factors such as the contamination level, pathogens’ persistence in water bodies, biological reservoirs (including aquatic plants and sediments) and the ability of pathogens to be transported [33]. The land use management practices and the size of the watershed also influence the survival of microorganisms [34,35,36]. According to George *et al.* [37], streams flowing through areas partly or fully covered with pastures are more contaminated than those running through forests and cultivated areas.

### 2.1. Fate in Sediments and in Submerged Aquatic Vegetation

Sediments and submerged aquatic vegetation (SAV) are important reservoirs of microorganisms [18,30,38]. For example, Badgley *et al*. [18] have shown that SAV harbored significantly higher mean densities of *Enterococci* than sediments, which themselves harbored higher densities than water. The high *Enterococci* densities observed in SAV are mainly due to increase nutrient availability as well as protection from UV radiation. Sediments can contain 10-times more viruses than water [33]. The microorganisms survival in different sediment types with different particle size and organic carbon content have been studied by Garzio-Hadzick *et al.* [39]. Their results revealed that sediments with small particle size and high organic carbon content could extended pathogens’ survival. Chandran *et al.* [40] concluded that sediments could act as a reservoir of pathogenic bacteria and exhibit a potential health hazards from possible resuspension and subsequent ingestion during recreational activities. The role of granulometric distribution of particles for microorganisms’ survival is also important for their transport during high flow events.

The behavior of microorganisms is also influenced by hydrometeorological changes such as heavy rainfalls which are likely to increase with climate change [12]. Thus, microorganisms associated with sediments or SAV, are put back into suspension and could lead to water contamination [18,33]. Re-suspension of sediments during or shortly after rainfall events was studied by Cho *et al.* [41] in order to investigate *E. coli* behavior regarding different bottom sediment textures of three streams for artificial high-flow events. A bacteria transport model was proposed, but additional research was needed to understand which and how sediment properties affect the parameters of streambed *E. coli* released into the water column.

### 2.2. Transport and Fate of Microorganisms

The associations between microorganisms and sediments influence their survival but also their transport characteristics [42]. Moreover the fate and transport of faecal bacteria are highly related to the governing sediment transport processes [38]. The transport of viruses linked to particles is particularly influenced by attachment to mineral surfaces and inactivation [43]. This adsorption can be reversible under other environmental conditions such as pH variations [44]. The microbial partitioning between the freely suspended and particulate attached phases during transport along overland flow pathways was studied by Soupir and Mostaghimi [45]. Rainfall simulations conducted on large-scale field plots showed that the majority of *E. coli* and *Enterococci* are transported from the fresh manure source in an unattached state, with only 4.8% of *E. coli* and 13% of *Enterococci* associated with particles. The average percent of attached *E. coli* or *Enterococci* is less than 3% with sparsely vegetated cover. In a laboratory-scale model system developed to investigate the transport mechanisms of *E. coli* in overland flow across saturated soils, *E. coli* appeared to be attached predominantly to small particles (<2 μm) and hence remained unattenuated during transport [45]. Moreover, microorganisms could be considered in hydric resources as biological particles transported by advection [33]. For example, Bekhit *et al.* [46] were interested in the role of colloids on the mobility of microorganisms and micropollutants in groundwater. They proposed a conceptual model to take into account the different physiochemical and biological processes, reaction kinetics, and transport mechanisms of the combined system (contaminant–colloids–bacteria). Different results have been obtained for natural aquatic media such as lakes and reservoirs. In particular, a review of Brookes *et al.* [47] explained the factors controlling pathogens transport and distribution, the respective role of dispersion, dilution and horizontal and vertical transport in such media. Contrary to lab-scale models, settling of pathogens particles operates with more complex hydrodynamic processes. Their results showed that all of the tested microbial indicators *(Escherichia coli*, *Enterococci*, *Clostridium perfringens*, aerobic spores, somatic coliphages, *Cryptosporidium* spp. and *Giardia* spp.) were associated with larger sized particle (>63.3 μm), except *C. perfringens* spores which were associated with particles size in the range of 45.5−63.3 μm. This granulometric distribution of the associated particles has important effects on the retention of microorganisms by settling and transfer into sediments [48,49]. Abudalo *et al*. [50] and Searcy *et al*. [51] precised that the environmental impact of protozoan parasites is closely related to their extended survival in contrasting climatic conditions and disinfection processes, and to their ability to interact with other organic or nonorganic particles [52]. The latter phenomenon governs their transport, retention (by settling) and/or release, and survival in the transition from land to sea.

In addition to Auer and Niehaus [53], Ferguson *et al*. [54], Sinton *et al*. [55] and Noble *et al.* [56] suggest that fecal indicator bacteria (FIB) inactivation rates (also referred to as a “die-off” or “decay” rate) vary under different environmental conditions, including solar radiation and water temperature. The FIB inactivation rate variability in response to other factors, such as water column depth, is not as well-understood, and has been recommended as an area for future research [57]. 

### 2.3. Influence of Hydrometeorological Conditions

Microorganisms’ transportation is particularly influenced by hydrometeorological conditions. Indeed microbial contamination of surface water increases during heavy rainfalls with water combined sewage overflows (CSOs) or agricultural land run-offs [33,58]. Rainfall increases the suspended matter content of small streams as well as their fecal contamination, with fecal coliforms mainly adsorbed on particles [37]. Rainfall events can also increase human enteric virus load in natural waters impacted by CSOs and stormwater leading to higher risk of gastroenteritis in recreational activities or shellfish consumption [59]. This risk has been evaluated using quantitative microbial risk assessment showing the dominance of norovirus in swimming-associated gastroenteritis [60]. Heavy rainfalls may also led to catchment area flooding with a strong increase of water turbidity, this last being considered as a non-questionable indicator of potential contamination by aquatic pathogens [61,62]. Several prospective epidemiological studies in France have also shown that groundwater influenced by surface water could be the cause of gastrointestinal infections in an endemic level [61,63], especially during rainfall events in karstic environment.

**Figure 1:**
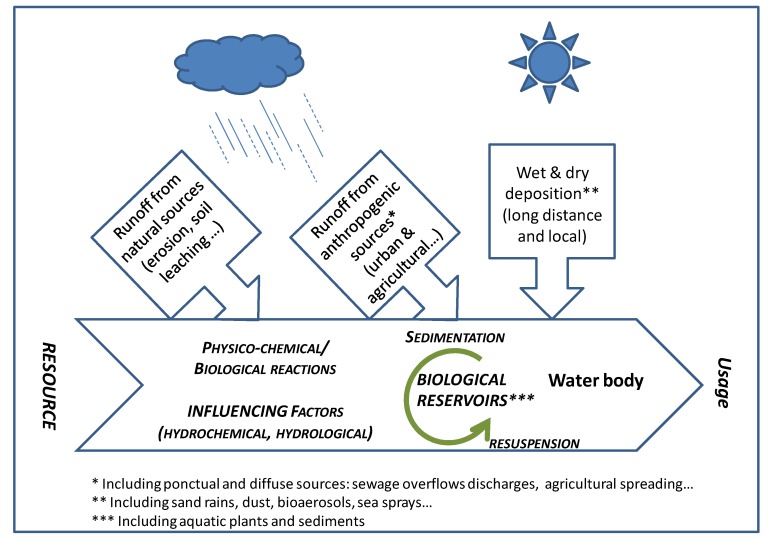
Schematic synthesis of source and fate of allochtonous microorganisms in water.

Water temperature variation plays also a role in the hydrodynamic distribution of microorganisms. In temperate areas, lakes are generally stratified during summer (with warmer water at the surface). Destratification occurs with heavy rains or storms, modifying the convection movements of particles containing microorganisms, moving them to the surface [64,65,66]. In a recent work, Wyer *et al*. [66] studied the variation of fecal indicators in four streams during a moderate rainfall. They demonstrated, thanks to microbial source tracking (MST), that the transfer time varies from minutes to hours, according to the hydrological conditions and sites characteristics. Table 1 presents some relevant studies dealing with pathogen interactions with particulate and colloidal phases in the aquatic compartment. Finally, Figure 1 proposes a schematic synthesis of source and fate of allochtonous microorganisms in water. From the resource to usages, the human pressure increases with production of drinking water and public use of recreational waters. From sedimentation to resuspension, the biological reservoirs are submitted to physicochemical and biological reactions on the one hand, and to hydrochemical and hydrological factors in water bodies on the other hand. Moreover, the external runoffs from anthropogenic and natural sources are considered as punctual and diffuse contribution, depending on climate change.

**Table 1:** Pathogens interactions studies with particulate and colloidal phases in the aquatic compartment.

*Type of pathogens*	*Matrix*	*Water Body*	*References*	*Comments*
*Bacteria*	Particles	Karstic aquifer	[67]	Connections with the surface responsible for turbid and bacterial contaminations
*Bacteria groups*	Colloids	Groundwater	[46]	Modelisation of transport mechanisms of the combined system (contaminant–colloids–bacteria)
		Lakes	[47]	Correlation between size particles and transport and distribution after a storm
		Recreational waters	[66]	Connection between microbial tracers and fecal indicator organisms
*E. coli, Enterococci*	Particles	Run-off	[68]	Rainfall simulations for erodible soil particles and sparsely vegetable soils
			[45]	run-off
*E. coli*	Sediments	Rivers	[41]	Modelisation of bacteria transport during rainfall events
*Virus* *Norovirus*	Colloids	Rivers	[69]	Direct spillage of wastewater in river during heavy rains
*Mixture*	Colloids	Distributed water	[3]	Correlation of heavy rains with gastroenteritis epidemics
	Particles	River, karstic water	[17]	Correlation of turbidity, flow rate and gastroenteritis epidemics
*Others* *Giardia cyst* *Protozoan parasites groups*	Colloids	Rivers	[70]	Correlation with rainy events
	Particles	Waterbeds soils	[52]	Interaction between parasites and particles (organic and inorganic)

As a consequence, the demand for faster and reliable monitoring methods and approaches is also increasing, with for example rapid molecular methods, although these methods do not allow a real-time control, contrary to optical methods [24].

## 3. Optical Monitoring of Microbial Contamination: Current Methods, Trends and Needs

### 3.1. Current Trends: Turbidity, Particle Size Distribution (PSD) and Cytometry

The main simple and practical way to assess the microbiological risk of water contamination is turbidimetry, largely used in water treatment plants. Despite its lack of sensitivity (there is no direct relation between pathogens density in water and turbidity), the choice of this parameter is relevant because: (i) the suspended particles are a safe and supportive environment for pathogens and a place of protection against disinfectants; (ii) turbidity lowers the efficiency of disinfection processes by the presence of oxidisable associated organic matter; (iii) turbidity in the water network creates particle deposition and promotes the growth of biofilms with the potential presence of pathogens (*Legionella*, *Pseudomonas*, *Aeromonas*, *Mycobacterium,* *etc*.) [17,71]. For 10 years, the interest in using turbidity for the continuous monitoring of effluents quality has been widely demonstrated [72,73,74]. In a constant environment, the respect for strict procedures gives precise and reliable turbidity data, in the laboratory as well as *in situ* [75], with a high frequency acquisition (minute order). Moreover, if this parameter is known as a good indicator of suspended matters in sewage networks [76,77,78], the turbidity measurement of fresh waters is influenced by the particle size and shape of SS, the presence of plankton and the presence of dissolved humic substances. Some authors have tried to establish correlations between turbidity and parameters like water level, flow rate, or rain intensity [73,74] without success, excepted in the case of a dilution phenomenon following a short dry period before raining [79]. Pronk *et al*. [80] have studied the dynamics of organic carbon (OC), turbidity, faecal indicator bacteria and physicochemical parameters in a karst system near Yverdon, Switzerland. OC appears to be a better indicator for bacterial contamination than turbidity. Page *et al.* [81] have tried to correlate continuous physico-chemical measurements (temperature, electrical conductivity, turbidity, spectral absorption coefficient (SAC), and particle density) with the amount of faecal indicator bacteria, such as *Escherichia coli* and *Enterococcus* *sp*., discontinuously sampled in the aquatic media. No individual proxy indicator for bacterial contamination was found. However, the following of the river water infiltration could be assessed by electrical conductivity, temperature and SAC measurements. 

Turbidity measurements are also often used for drinking water quality monitoring and within water treatment plants considering experimental approximate relations with the number of germs [82]. It is regulated by the Directive 98/83/CE for the distributed waters but not for resources, contrary to microbiological parameters considered in both cases. Even if turbidity measurements are often used as a run-off pollution indicator of resource water, the precise interpretation of this parameter as a sanitary threat indicator, remains difficult because of its sensibility to environmental factors as described before. Beaudeau *et al.* [17] have recently shown a significant and biologically plausible risk was highlighted on rivers. Turbidity of treated water may be considered as an exposure indicator which produces the more reproducible risks [71]. 

The physico-chemical characterization methods of particles (shape, size, surface properties) were recently considered in particular because of the sensitivity of turbidity to these parameters. Among them, Goldscheider *et al.* [83] used a portable field particles counter in order to explain the turbidity measurements by following the temporal evolution of particle size distribution (PSD) between 0.9 and 139 µm diameter. For karstic resources, turbidity measurements fail to accurately estimate microbiological contamination because of the variability of the hydroclimatic conditions and the presence of more or less large particles, remobilized by the first flush, followed by a very fine particle mix (about 1 µm), sometimes with the presence of pathogens coming from contaminated waters, confirming the results of Atteia and Kozel [84]. A very good correlation between turbidity and *E. coli* (R^2^ = 0.93) has been demonstrated for colloidal particles (0.9–1.5 µm).

Cytometry techniques are also based on optical principles and are now routinely used in microbiology, particularly for microbial ecology studies, unfortunately not on site. Flow cytometry can detect microorganisms at the cellular level from their optical properties (fluorescence and light scattering), to quantify and provide information on the individual characteristics of cells and information on the heterogeneity of cellular states in a population. It applies to the study of autotrophic and heterotrophic microorganisms the fluorescence properties of which are natural (pigments) or induced by probes targeting taxonomic targets and/or physiological. Particles analyzed in the environmental field are diverse and range from viruses to protozoa. The analysis time is less than 1 h and the number of cells analyzed is very high (around 10^7^ colony forming unit/mL). In some cases it is possible to physically separate the cell functions of some of their optical properties in order to complete their analysis by other techniques (biochemical and molecular biology techniques) [85,86].

### 3.2. Trends in Optical Methods

#### 3.2.1. Fluorescence Measurements

Although fluorescence measurements are well known for organics analysis (e.g., hydrocarbons), this method could also be considered for its interest in microbial sources tracking. The origin and contamination type of dissolved organic matter (DOM) has been characterized by fluorescence [87,88,89]. For example, in river water samples, both protein-like (tyrosine- and tryptophan-like) and fulvic-like and humic-like fluorescence were detected and the relative intensity of these signals was used as an indicator of slurry occurrence in drainage waters in agricultural area [89]. Jaffrezic *et al.* [90] propose the geochemical fluorescence index (GFI), to point out the difference between farm waste and human waste. GFI is the ratio between two regions of the fluorescence spectrum divided into biochemical and geochemical regions. Lefcourt *et al*. [91] have developed a field-portable fluorescence imagining system for detecting fecal contaminants for agricultural products.

Exploitation of the bacteriophage (or phage) life cycle and its accompanying species specificity can reduce much of the time and cost associated to classical methods such as gene sequencing, electrophoresis or repetitive sequence-based polymerase chain reaction (PCR). The well-characterized bacterial luciferase genes, encoded by the lux operon from the aquatic Gram-negative *Vibrio fischeri* are perhaps the most frequently used reporters for incorporation into recombinant phages and have many applications, namely for acute toxicity prediction for chemicals [92]. Other-component bacteriophage-based bioluminescent reporter systems were developed for the detection of *Escherichia coli* in environmental samples, for instance [93].

#### 3.2.2. Biosensors

Biosensors combining the selectivity of biology with the processing power of microelectronics may also be considered through bio-recognition systems, generally enzymes or binding proteins such as immobilized antibodies. Biosensors have the potential for substantial improvements over standard methods and have been reported employing the full spectrum of biorecognition molecules and transduction methods for detection of waterborne pathogens, with oligonucleotide probes and antibodies being the most common [94]. These biosensors have been used in various fields such as environment or food processing for biorecognition of specific molecules, since they have a high sensitive detection capacity. However, several problems for waterborne pathogens detection may appear with regard to low concentration and many interfering enzymatic reactions in natural media [94]. In the literature, many examples for the detection of *E. coli* can be found, and among these studies, the transduction based methods, with fluorescence and chemiluminescence probes are the most used [94]. (Bio)sensing systems have benefited from the achievements of scientific and technological research from nanomaterials and nanotechnologies science [95]. Especially, nanoparticles, gold nanoparticles, graphene, quantum dots, have led to major advances in this field [96]. Various mass-sensitive techniques have also been applied for bacteria [94]. For these techniques, the limits of detection are around 100 cells/mL. Others techniques using nanocrystal biosensors are used for drinking water market, since they seem to be reliable and are *in-situ* techniques for monitoring multiple pathogens mixtures. Alternatively, many studies concerning protozoan biosensing detection can be related since the detection schemes applied to parasites and viruses too [94].

#### 3.2.3. Spectrophotometric Methods

Spectrophotometric methods are potentially interesting for the detection of microbial contamination because of their use in particle characterization. As shown in the review and synthesis of Bowers and Binding [97], spectrophotometric methods are used for quantitative assessment of particle concentration in sea water, in order to study the effect on sunlight penetration into the sea. Astoreca *et al.* [98] have correlated the basic properties of the particles (concentration, composition, and size of suspended particles in sea water) with their light absorption properties in the visible and near-infrared regions. Stramski *et al*. [99] have proposed a database of the single-particle optical properties of marine microbial particles to better understand the ocean optics. This database (based on the optical properties depending on the particle size) includes representatives from five classes of particles: viruses, heterotrophic bacteria, cyanobacteria, small nanoplanktonic diatoms, and nanoplanktonic chlorophytes. More recently, Stadler *et al*. [100] have obtained good correlation between SAC_254_ (spectral absorption coefficient at 254 nm) and *E. coli* in karst water resources and propose that SAC_254_ may also be used as a real-time proxy to estimate the magnitude of faecal pollution during rainfall events. They precise that only diffuse faecal pollution sources with adequate soil contact are supposed to be quantifiable by the described approach.

Enlarging this approach, UV/Visible spectroscopy has lots of advantages to study the interactions between natural matrices and pollutants in dissolved and colloidal compartments [101]. The first advantage of this technique is to be rapid, direct and low cost. Several studies have proven the usefulness of such a technique in water quality monitoring, especially thanks to the complementarities with basic analysis such as dissolved biodegradable organic carbon [102], total organic carbon [103], by developing spectral exploitation techniques such as deconvolution or multi-wavelength treatment [104,105]. Owing to its good sensitivity, this technique could be useful to provide semi-quantitative information on the interactions between phases (organic carbon exchange) by direct monitoring, a non-specific pre-analysis preparation and at natural media concentrations. 

**Table 2:** Characterization of particles by UV (water and wastewater).

References	Optical domains	Measurement/study	Particle size (µm)	Suspended matter concentration (mg/L)
[98]	Visible and near-infrared spectral regions	Relationships between the concentration, composition and size of suspended particles	2.72–460	0–90
[106]	UV spectrophotometry and laser granulometry	Characterization of heterogeneous suspensions	0.4–2 × 10^3^	100–670
[107]	Coupling UV-spectrophotometry and laser granulometry	Heterogeneous suspensions, quantitative approach (size and concentration)	0.05–10^3^	10–350
[108]	UV spectrophotometry	Study of the impact of mechanical treatments on wastewater solids by UV spectrophotometry	10^−3^–10^3^	10–220
[109]	UV spectrophotometry and laser granulometry	Study of UV–vis responses of mineral suspensions in water	1–100	10–250

The technique could also give information about compounds’ size by coupling UV/Visible spectroscopy to laser granulometry analysis [106,107,108,109]. Behro *et al.* [109] proposed a typology of UV responses of particles according to their size and a model spectrum of mineral colloids in order to obtain a better understanding of physical absorption phenomena. They showed that the UV responses of non-absorbing particles can be modeled even if the physical phenomena are complex and the refraction index and the shape of particles have not been taken into account.

Table 2 synthesizes some results obtained by spectrophotometry for the characterization of particles in heterogeneous suspensions including water and wastewater. Table 3 presents the main literature studies presented in Section 3.2.2 of the manuscript.

## 4. Conclusions

This review has shown that knowledge about pathogen transport mechanisms are partial, even if the role of particles and colloids is relatively well documented. Moreover there is a lack of methods (index, sensor) allowing onsite identification of microbial contaminants and dynamic exchanges of pathogens into different phases such as soluble, colloidal, and particulate. Concerning the first point, the improvement of the optical responses currently provided by turbidimetry could be an interesting solution to identify hazardous situations of water microbial contamination. Indeed, the pertinence of the turbidity measurement is limited, since nephelometric measurements are dependent on the presence of colloids that may interfere with the results. 

**Table 3:** Synthesis of classical and trends in optical methods for pathogens detection.

Parameter/References	Kind of media/applications fields/pathogens	Influencing parameters for the studies/interferences	Particle size/Number of celldetected
Turbidity/[7]–[72,73,74,75,76,77,78,79,80]	Natural and wastewaters	Plankton, Humic substances	10–10^3^ µm
PSD/[3,84]	Karstic waters	Hydroclimatical	0.9–1.5 µm
Cytometry/[85,86]	All fluorescent species	Others fluorescent species (e.g., humic-like substances) + light scattering	From virus to bacteria/10^7^ colony forming unit/mL
Fluorescence, Bacteriophage life cycle/[87,88,89,90,91,92,93]	River waters (tyrosine, tryptophan and fulvic-like substances, *E. Coli*, *Vibrio fischeri*	Light-scattering, inner filters effects, bioluminescence interferences	From molecule to bacteria
Biosensors/[94,95,96]	Environment, food process, military	Interfering enzyme reactions	Virus to protozoan> 100 cells/mL
Spectrophotometry Methods/[97,98,99,100,101,102,103,104,105,106,107,108,109]	Virus, bacteria, cyanobacteria, nanoplanktonic and chlorophytes diatoms	Light scattering	10^−3^–2 × 10^3^ µm

* to have a correlation between the size and the measurement value.

Among the ways of improvement encountered, the relevance of UV-Visible spectra exploitation to obtain significant information about the chemical and granulometric composition of the particulate phase is underlined. Thus, the development of a simple physico-chemical proxy (UV visible exploitation) associated to other optical methods (particle size distribution, turbidimetry, fluorimetry…) should be a research path to obtain an indicator for hazardous situations. 

For the second point, the development of a characterization procedure (static and dynamic) allowing the study of the exchanges between particles and the microbial agents, namely during heavy rainfall events (aggregates, resuspension…) is needed. The exchanges being related to the size, the concentration, and particles charges and nature, all these parameters should be considered. Moreover, different operational conditions (e.g., pH, conductivity, organic carbon concentration) must be also assessed to improve the knowledge of the matrix effect (environmental hazardous situations). As shown by the review, there is a real lack of temporal studies integrating the seasonal and hydrological parameters associated to heavy rainfall events with regard to health risk assessment.

All these research needs must be considered at fair value because of, in addition to the population health impact, the importance of cost management of waterborne diseases such as acute gastroenteritis is undeniable. The cost reduction, by a better anticipation of the sanitary degradation of natural water resources quality, is therefore an important economical issue. For the scientific point of view, the main question will be to complete the main exposure indicator (the turbidity measurement) with additional more specific parameters, especially hydrological conditions. This needs a multidisciplinary approach between hydrologists, chemists, microbiologists, water treatment managers and epidemiologists. 

The outcomes of these studies could give arguments to water treatment managers to modify or adapt the water treatment processes, or a different way to manage their plants by taking into account an alert procedure proposed for hazardous situations, based on new indicators of microbial contamination.
